# Combinatorial design of a sialic acid imprinted binding site exploring a dual ion receptor approach†

**DOI:** 10.1039/d1ra06962d

**Published:** 2021-10-22

**Authors:** Liliia Mavliutova, Elena Verduci, Börje Sellergren

**Affiliations:** Department of Biomedical Sciences, Faculty of Health and Society, Malmö University SE-20506 Malmö Sweden borje.sellergren@mau.se

## Abstract

Aberrant sialic acid expression is one of the key indicators of pathological processes. This acidic saccharide is overexpressed in tumor cells and is a potent biomarker. Development of specific capture tools for various sialylated targets is an important step for early cancer diagnosis. However, sialic acid recognition by synthetic hosts is often complicated due to the competition for the anion binding by their counterions, such as Na^+^ and K^+^. Here we report on the design of a sialic acid receptor *via* simultaneous recognition of both the anion and cation of the target analyte. The polymeric receptor was produced using neutral (thio)urea and crown ether based monomers for simultaneous complexation of sialic acid's carboxylate group and its countercation. Thiourea and urea based functional monomers were tested both in solution by ^1^H NMR titration and in a polymer matrix system for their ability to complex the sodium salt of sialic acid alone and in the presence of crown ether. Combination of both orthogonally acting monomers resulted in higher affinities for the template in organic solvent media. The imprinted polymers displayed enhanced sialic acid recognition driven to a significant extent by the addition of the macrocyclic cation host. The effect of various counterions and solvent systems on the binding affinities is reported. Binding of K^+^, Na^+^ and NH_4_^+^ salts of sialic acid exceeded the uptake of bulky lipophilic salts. Polymers imprinted with sialic or glucuronic acids displayed a preference for their corresponding templates and showed a promising enrichment of sialylated peptides from the tryptic digest of glycoprotein bovine fetuin.

## Abstract 2

Dual-ion imprinting of sialic acid *via* cooperatively acting ureido- and crown ether functionalities leads to charge neutral sialic acid receptors with strong sialoglycopeptide affinity.

## Graphical abstract

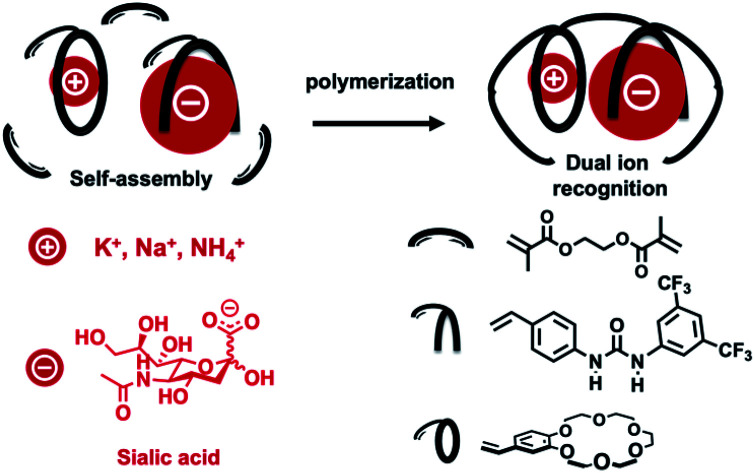

## Introduction

Sialic acids (SAs) are typically found as terminal moieties of glycans and play a major role in various biological processes.^1^ SAs consist of a variety of structural analogs and are shown to be potent biomarkers for diseases such as cancer.^2–4^ SA analysis is usually performed with the help of natural receptors, such as lectins and antibodies, as well as using general enrichment phases, such as metal affinity or hydrophilic interaction chromatography (HILIC).^5^ Molecularly imprinted polymers (MIPs) offer an alternative for the selective binding of sialylated targets.^6–8^ This technique produces robust synthetic receptors *via* polymerization of functional monomers and crosslinkers in the presence of a template molecule.^9^ Subsequent template removal results in binding sites that are specific for the template. Various functional monomers have been applied for SA imprinting, relying on interactions of boronic acid monomers with the *cis*-diol groups of SA^6,10,11^ and/or positively charged monomers with SA's carboxylate.^12,13^ Moreover, hydrogen bonding host monomers containing charge neutral urea binding motifs^14^ have been employed in the imprinting of acidic saccharides, such as SA and glucuronic acids (GA) in combination with boronic acid and/or amine-based co-monomers to improve binding in highly competitive aqueous environment.^8,15^

As for urea-based low-molecular anion hosts,^16^ these are designed to bind the anion only, thus leading to energetic penalty caused by the unfavorable separation of the ion pair. Therefore, the majority of the anion binding hosts use salt systems with non-competing counterions, such as tetrabutylammonium (TBA) or 1,2,2,6,6-pentamethylpiperidine (PMP), for binding to take place.^17^ Carboxylate group featuring high charge density and strong hydration is a hard anion, compared to phosphate and sulphonate, and interact strongly with hard counterions like Li^+^, Na^+^, K^+^.^18^ Thus, SA binding by neutral hosts is susceptible to competition by alkali metal cations, hindering their application on a broader scale.

**Fig. 1:**
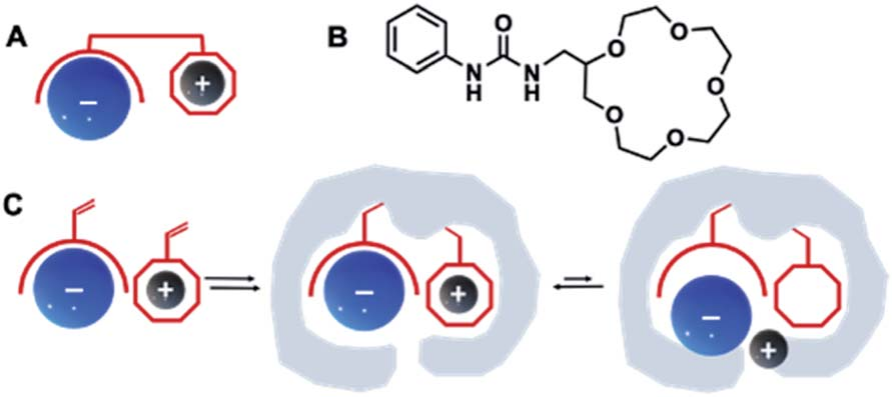
Schematic illustration of ditopic receptor design for ion recognition (A) with an example of urea/crown ether receptor (B),^17^ and dual ion imprinting strategy (C).

Ion-pair recognition, in which a host simultaneously binds both cationic and anionic species, offers a solution to overcome this problem. In so called ditopic binding, the cations and anions are typically bound as a separated or contact ion pairs (Fig. 1A) through anion-binding functionalities, such as urea, and cation-binding groups (*e.g.*, calixarene or crown ether).^19^ Such multisite frameworks often exhibit cooperative or allosteric behavior, where binding of one ion enhances the binding of the other.^20,21^ The heteroditopic receptors designed by Smith *et al.*^17^ combined urea and crown ether groups for ditopic binding of various anion–cation pairs (Fig. 1B). Another type of ditopic receptors reported by Ungaro *et al.*^22^ includes calixarene and (thio)urea functionalities and are capable of simultaneous complexation of cations and carboxylate anions. However, synthesis of such receptors is often cumbersome and requires careful design to match anion–cation geometries to achieve selectivities for a certain target.

One direct way to construct such receptors is *via* dual ion imprinting approach by combining macrocyclic cation hosts with neutral urea-based anion receptors (Fig. 1C). A successful example of such technique has been previously reported for the imprinting of the disodium salt of phenyl-phosphonic acid in combination with neutral diaryl urea and 4-vinylbenzo-18-crown-6 (VB18C6) monomers.^23^ The polymers displayed enhanced affinities for their template, retained in competitive aqueous buffers and high salt media. Encouraged by these results, we decided to expand our previously reported combinatorial approach^15^ to produce imprinted receptors with enhanced recognition of Na^+^, K^+^ and NH_4_^+^ salts of SA. In the present study, the effect of (thio)urea and 18-crown-6 monomers on recognition properties of SA receptors was studied. Molecularly imprinted polymers were synthesized as microparticles and used as neutral capture phases for the enrichment of sialylated glycans/glycopeptides from a model protein digest.

## Results and discussions

### Study of complex formation in solution

Design of the imprinted polymers typically starts with the selection of optimal functional monomers capable of forming strong interactions with the template. A neutral receptor for SA has been described by Figueroa *et al.*,^24^ which combines a strong bidentate hydrogen bonding motif of thiourea with boronate ester formation on carboxylate and glycerol moieties of SA, respectively. They showed that thioureido functionality was a stronger binder for SA in comparison to ureido group. It is expected that the more acidic thiourea containing receptors are able to form more stable complexes with anions than their urea-containing counterparts (p*K*_a_ = 21.1 and 26.9, respectively in DMSO).^25^ Thus, they might be suitable for recognition of more acidic targets, such as SA (p*K*_a_ = 2.6).

**Fig. 2:**
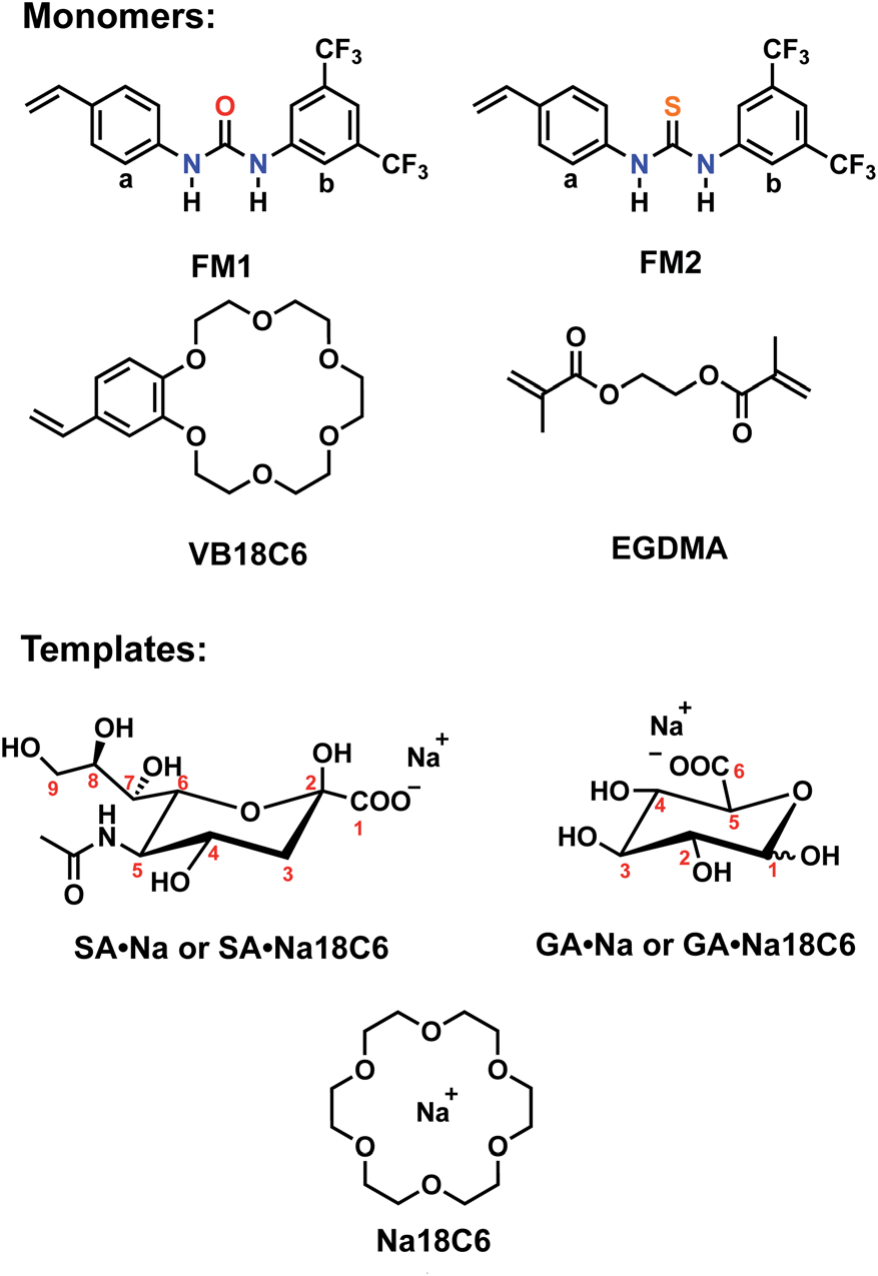
Structures of functional monomers and templates with corresponding counterions.

**Fig. 3:**
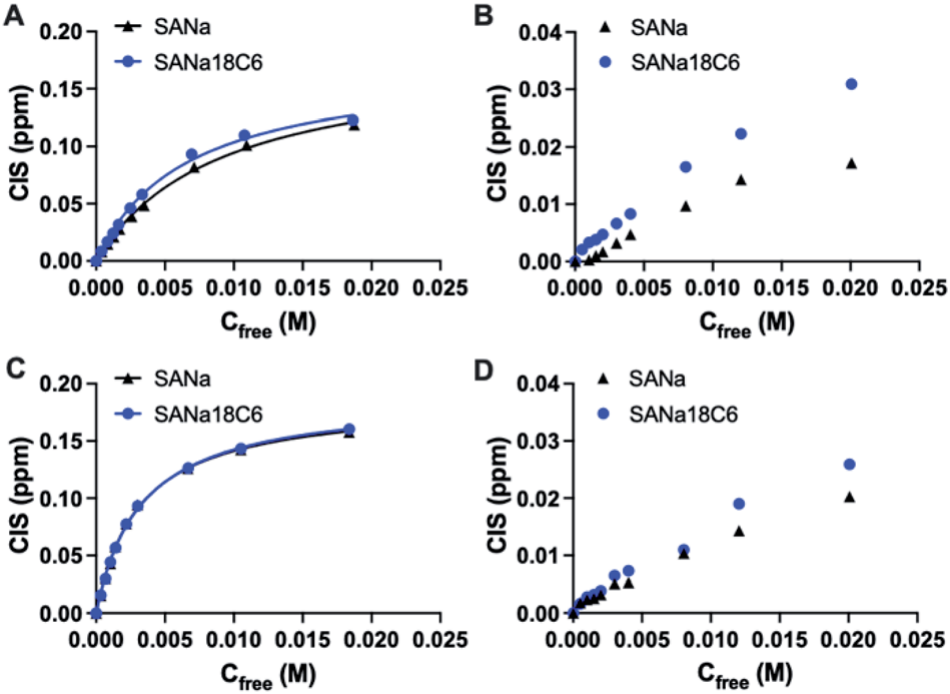
Complexation induced shift of H_a_ protons upon titration of FM1 (A and B) and FM2 (C and D) with SA·Na and SA·Na18C6 in DMSO-d_6_ and CD_3_OD, respectively. Curves are fitted with mono-Langmuir binding model.

Structure of the monomers and templates used in this study are shown in Fig. 2. Ureido (FM1) and thioureido (FM2) monomers were compared for their ability to form complexes with sodium salts of sialic (SA·Na) and glucuronic (GA·Na) acids in DMSO-d_6_ and CD_3_OD. The effect of macrocyclic cation host 18-crown-6 (18C6) on the complex stability was also investigated by means of ^1^H NMR titration studies. Complexation induced shifts (CIS) of relevant protons were monitored and plotted against free guest concentration (*C*_free_). Resulting binding isotherms were used to derive the binding coefficients (Fig. 3). Titration of FM1 with SA·Na solution in DMSO-d_6_ resulted in a clear downfield shift of the urea protons NH (Fig. S1†). The binding curve, fitted to one-site Langmuir binding isotherm, gave an association constant of 111 ± 7 M^−1^ (Table 1). In the case of FM2, the thiourea protons were fully deprotonated after addition of 1 eq. of guest, indicated by disappearance of the NH resonance (Fig. S2†), so the binding constant were determined by observing CH protons on 1,3-aryl substituents (H_a_ and H_b_). Thiourea containing receptors are prone to deprotonation in the presence of anions in the following order F^−^ > CH_3_COO^−^ > C_6_H_5_COO^−^ > H_2_PO_4_^−^, depending on the stability of anion self-complex.^26^ Deprotonation occurs on the most acidic NH proton, according to NMR data with broadening of the CH_b_ resonances in the course of titration. The host's aromatic protons on the –Ph(CF_3_)_2_ moiety shift considerably accompanied by broadening, indicating possible rearrangement/interconversion of conformation and slow exchange of free and complexed guests on the NMR time scale. Nonetheless, thiourea monomer FM2 displayed a 3-fold increase in binding constants for SA·Na in comparison to its urea-based counterpart in aprotic DMSO-d_6_ (Table 1). Both FM1 and FM2 displayed higher affinities towards more basic GA·Na (p*K*_a_ = 3.2). Deprotonation of urea protons was observed for FM2 in this case as well (Fig. S3†).

**Table 1:** Binding constants and complexation induced shifts^a^ for complexes formed between (thio)urea monomers and SA·Na/SA·Na18C6/GA·Na/GA·Na18C6 in DMSO-d_6_ at 25 °C

Host	Guest	*K*_a_, M^−1^	CIS, ppm	*R* ^2^
FM1	SA·Na	111 ± 7	0.198	0.9983
FM2	SA·Na	321 ± 13	0.185	0.9985
FM1	GA·Na	361 ± 52	0.099	0.9815
FM2	GA·Na	368 ± 36	0.147	0.9914
FM1	SA·Na18C6	152 ± 13	0.193	0.9957
FM2	SA·Na18C6	305 ± 9	0.189	0.9992
FM1	GA·Na18C6	240 ± 31	0.114	0.9877
FM2	GA·Na18C6	456 ± 83	0.211	0.9683

a H_a_ protons monitored and fitted to one site specific binding isotherm.

The addition of 18C6 had opposite effect on the binding affinities of FM1 and FM2. Whereas *K*_a_ of thiourea slightly dropped, urea *K*_a_ increased upon addition of crown ether ligand. A similar trend was observed in CD_3_OD, with FM1 showing greater differences in the shifts in comparison to FM2 with and without 18C6. The lower CISs observed in case of FM2 + 18C6, most likely reflect the deprotonation of the thiourea based receptor in polar solvents. Although the *K*_a_ determination was not possible in this case, overall trends correspond to the ones observed in DMSO-d_6_. Affinity of FM1 towards GA·Na18C6 was slightly lower than in the case of simple GA·Na salt accompanied with more pronounced deprotonation of the urea protons (Fig. S4–S6†).

### Polymer synthesis and characterization

Imprinted polymers P1–P5 were prepared and characterized using the (thio)urea monomers FM1 or FM2 and crown ether monomer VB18C6 as listed in Table 2. Monomers were added in 1 : 1 equivalent to the SA·Na template and polymerized in presence of 20 equivalents of crosslinker EGDMA in methanol at 50 °C. Non-imprinted polymers (P_N_1–P_N_5) were prepared identically to the imprinted polymers but omitting the template from the prepolymerization mixture. A control polymer with GA·Na template was prepared as well. After polymerization, polymers were crushed and subjected to solvent extraction to remove templates.

**Table 2:** Imprinted polymer composition with equivalent of functional monomers, template (T) and crosslinker (X)^a^

Polymer	FM1	FM2	VB18C6	T	X
P1	1	—	—	1 (SA·Na)	20
P2	1	—	1	1 (SA·Na)	20
P3	—	1	—	1 (SA·Na)	20
P4	—	1	1	1 (SA·Na)	20
P5	—	—	1	1 (SA·Na)	20
P6	1	—	1	1 (GA·Na)	20

a NIP P_N_1–P_N_5 produced without template addition.

Characterization of the final materials by FTIR (Fig. S7–S10†) showed no apparent variations in chemical composition between MIP and NIP. However, scanning electron microscopy revealed differences in the morphologies between the polymers (Fig. S11–S15†). Urea-based SA·Na-MIPs P1/P2 and their corresponding NIPs P_N_1/P_N_2 displayed irregular particles with rough surfaces with no significant differences between imprinted and non-imprinted materials. In contrast, thiourea-based polymers P3/P4 and P_N_3/P_N_4 as well as VB18C6 polymers P5/P_N_5 show micron sized particles with irregular agglomerated structures for MIP and fine globular particles for the NIP. This is possibly related to the differences in the solubilities of the monomers FM1 and FM2, as well as effect of template complexation on the solubility of the growing polymer chains and subsequent onset of precipitation/delay in phase separation.

**Fig. 4:**
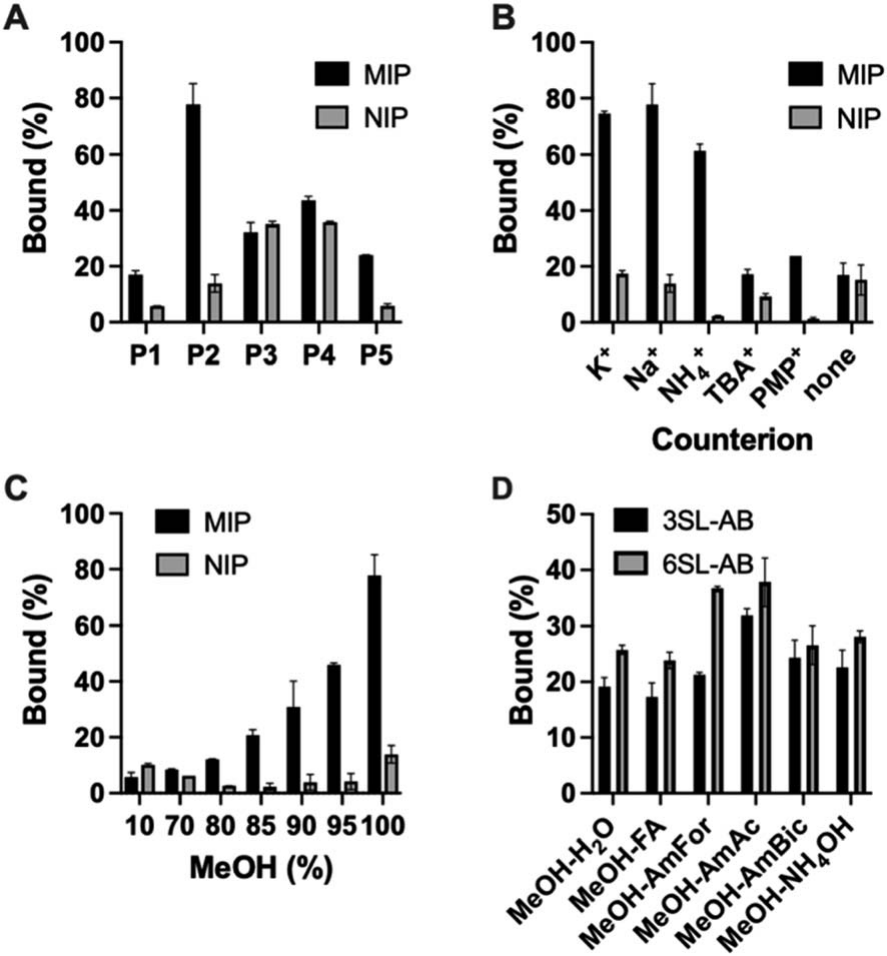
Uptake of 0.5 mM SA·Na by P1–P5/P_N_1–P_N_5 in 100% MeOH (A), counterion effect on binding of SA salts on P2 in 100% MeOH (B) effect of MeOH concentration on SA·Na binding (C). Binding of 0.05 mM equimolar mixture of 3SL-AB/5SL-AB by P2 in 80% MeOH in various buffers (D).

Polymers were then characterized for their ability to recognize the template in the batch rebinding experiments. Uptake of SA·Na by MIP and NIP was tested in 100% MeOH (Fig. 4A). The combination of urea monomer FM1 and VB18C6 in polymer P2 gave rise to an enhanced uptake of SA·Na with clear difference between imprinted and nonimprinted polymers. In comparison, polymers prepared with only one of the functional monomers, P1 and P5, exhibit lower template binding, but still retained the imprinting effect. The competitive nature of the solvent seemed to have less of an impact on the dual ion imprinted polymer. Thiourea-based polymer P3 shows overall higher uptake of template, but also displayed increased non-specific binding judged by the NIP performance. This is in agreement with NMR titration data, where thiourea monomer showed higher affinity constants in comparison to its urea counterpart. However, the imprinting effect was not as pronounced in this case even with the addition of crown ether moiety in P4. As we reasoned above, it is possible that methanol, used in the polymer preparation, promotes the deprotonation of FM2 thereby preventing formation of a defined binding site. Alternatively, the monomer may exist in a non-optimal conformation at the polymerization conditions (higher temperatures and concentrations). Focusing on the promising performance of the P2 polymer, effects of counterion and solvent composition on binding were studied in more detail.

Polymer P2 was characterized for its cation recognition abilities. Crown ethers are macrocyclic hosts able to complex metal ions with a 1 : 1 host–guest stoichiometry. The 18-membered ring macrocycle features a cavity size matching the abundant alkali cations K^+^ and Na^+^ (log *K* = 6.1 and 4.3 in MeOH).^27^ The complexation of ammonium compounds with crown ethers has also been reported with higher stability constants for the complexes formed in methanol (log *K* 1.44 in H_2_O, 4.22 in MeOH, 1.34 in DMSO for BzNH_3_Cl,^28^ and log *K* 2.0 in H_2_O, 4.32 in MeOH, 2.48 in DMSO for NH_4_^+^).^29^ This was explained by lower solvating strength of methanol leading to weaker competition with the polyether for the cations.^27^

The stability of the crown ether complexes depends primarily upon optimal size match of the macrocyclic ring with the cation, the charge density of the cation and the solvating power of the medium.^27^ In order to check the counterion effect, SA salts with different cations (K^+^, Na^+^, NH_4_^+^, TBA^+^, PMP^+^) were incubated with P2 in 100% MeOH (Fig. 4B). The uptake of the analyte with K^+^ and Na^+^ as counterions exceeded binding of all other salts proving the presence of ion-pair complementary sites and in line with macrocycle-cation size match. NH_4_^+^ has shown second best binding apart from alkali metal ions, in agreement with general affinity of ammonium for 18C6. Ammonium salts could be optimal candidates for glycomics experiments, being volatile and compatible with organic solvents. On the contrary, SA with bulky lipophilic TBA^+^ and PMP^+^ counterions did not bind strongly to the polymer, possibly due to steric constraint to fit the SA·Na imprinted cavity and lacking affinity to VB18C6. Expectedly, SA in free acid form did not bind to the polymer lacking activated carboxylate group and matching counterion.

Due to the crucial role of the solvent on urea's and crown ether's binding affinity, the effect of water content on binding was examined and shown in Fig. 4C. The drastic decrease in binding was observed at MeOH concentration below 85% (v/v). The loss of binding can be ascribed to three factors, (1) the disruption of hydrogen bonds between urea receptor and the analyte, (2) cavity collapse of VB18C6 upon exposure to water and (3) saccharide hydration effects. Therefore, the working range of the polymer is currently limited to high organic modifier content. Nevertheless, the conditions are compatible with typical procedures for glycopeptide and glycan enrichment and separation techniques, for example in standard HILIC setups.^30–33^ Binding in acetonitrile–water mixtures resulted in higher uptake of SA·Na comparing to methanol mixtures, most likely due to the weaker solvation of the saccharide in the acetonitrile rich media (Fig. S16†).

Optimal binding/elution conditions for glycan/glycopeptide enrichments were investigated using 2AB labelled sialyllactose trisaccharides as sialylated glycan targets. Various conditions such as 10 mM ammonium bicarbonate (AmBic, pH 8), acetate (AmAc, pH 6), formate (AmFor, pH 4) as well as 0.5% NH_4_OH (pH 10) and 0.5% FA (pH 2) in 80% MeOH were used as solvents in the equilibrium binding experiments using 50 μM equimolar mixture of 2AB-labelled sodium salts of sialyllactoses. Lower analyte binding was observed using highly acidic or basic conditions, such as NH_4_OH/AmBIC and FA solutions (Fig. 4D). Those conditions could be used for the elution step. Increased binding was observed using intermediate pH 4–6 with AmFor and AmAc buffers, with the higher uptake of 2,6′-sialic acid isomer. Those buffers have a carboxylate group that could compete for the imprinted sites. Alternatively, blocking of non-specific binding sites could be accomplished, reducing the unfavorable interactions. A higher aqueous content was used to accommodate the solubility limitations of glycans in organic solvents. Use of buffers with high ionic strength (50 mM) did not reduce the uptake of the SA·Na template molecule, instead it seemed to enhanced it (Fig. S17†).

**Fig. 5:**
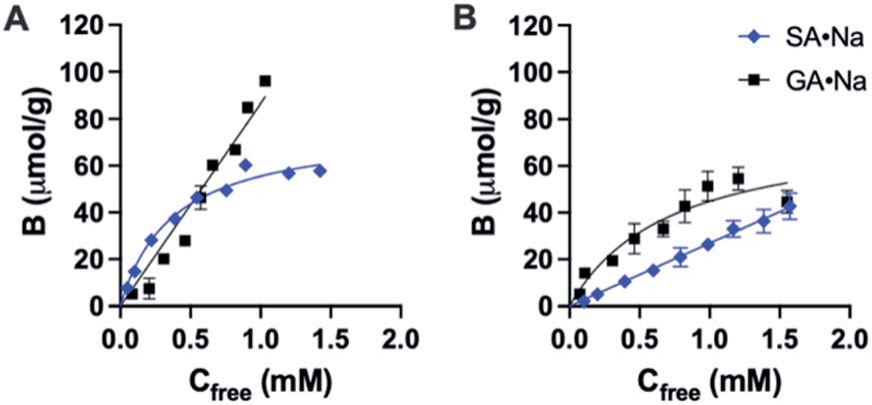
Binding isotherm of P2 (A) and P6 (B) with SA·Na and GA·Na in 95% MeOH, fitted to one site specific binding model.

The binding affinity and selectivity of P2 was tested by recording a binding isotherm in 95% MeOH with SA·Na and GA·Na. The imprinted polymer displayed selectivity towards its sugar acid with *K*_a_ = 2.7 × 10^3^ M^−1^ obtained from fitting to one site specific binding model (Fig. 5A and Table S1†). Although some level of cross-reactivity was observed with GA·Na, another sugar acid with slightly higher basicity (p*K*_a_ ≈ 3.2), this can be attributed to non-specific binding at high organic modifier content. As a control polymer, GA·Na imprinted polymer P6 was produced and tested for SA·Na and GA·Na rebinding. The polymer showed high preference for the template GA·Na with *K*_a_ = 1.4 × 10^3^ M^−1^ and low cross-reactivity with SA·Na (Fig. 5B and Table S2†). The NIP displayed lower uptake of both saccharides (Fig. S18†). Thiourea based polymers P3 displayed higher affinities towards SA·Na and negligible binding of more basic GA·Na. The binding curve of SA·Na on P3 was shallow and identical to that of the NIP indicating absence of imprinting (Fig. S19†). Slightly higher uptake of GA·Na was observed only in the case of P4, composed of both FM2 and VB18C6 monomers, reflecting the influence of crown ether functionality of the ion pair affinity. Overall, the results suggest that deprotonation of more acidic FM2 prevents the formation of well-defined imprinted binding sites in methanol.

### Glycopeptide enrichment

**Fig. 6:**
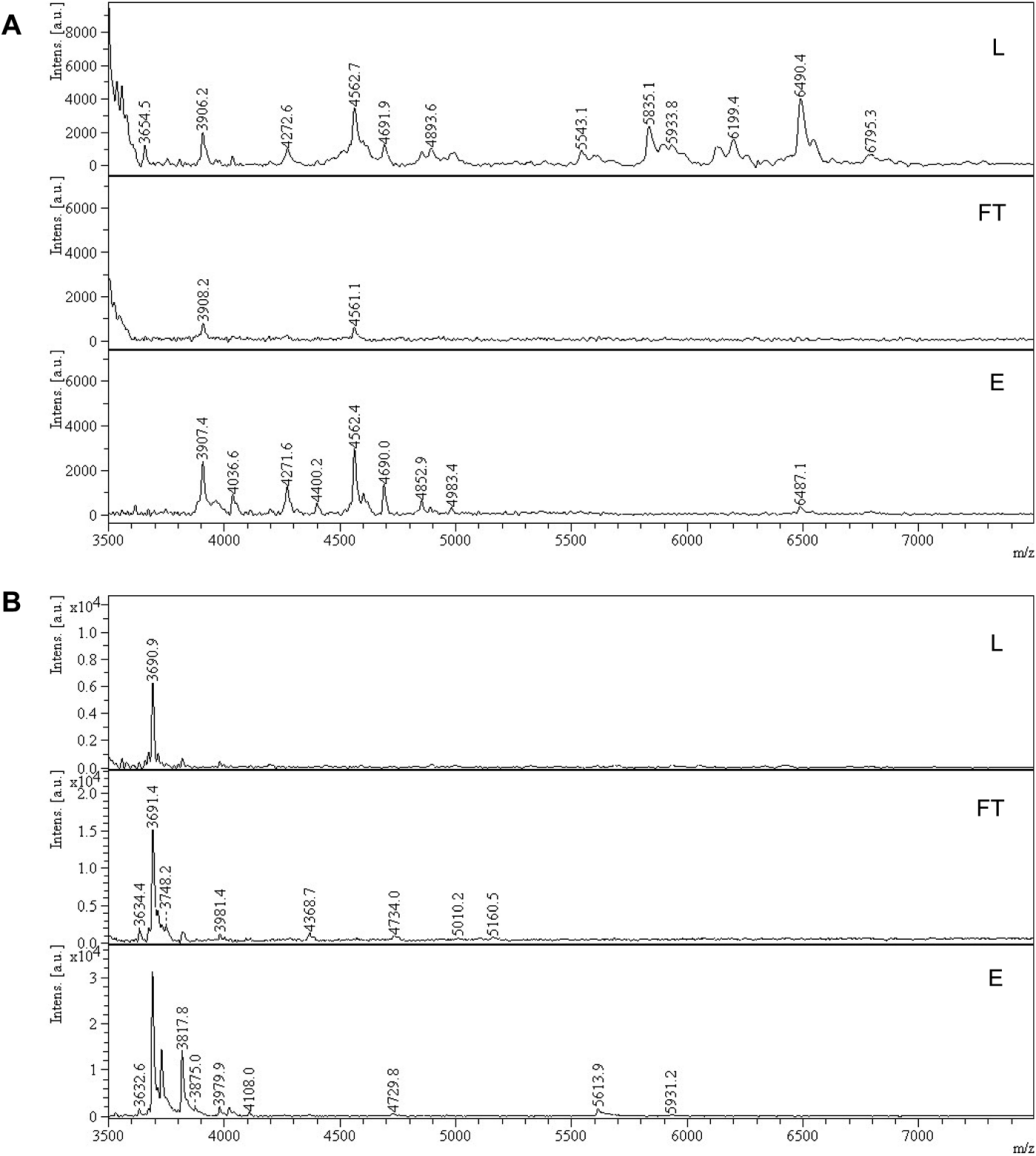
MALDI-TOF-MS of tryptic peptide mixtures of fetuin bovine (A) and sialidase treated fetuin bovine (B). Part of the peptide spectrum is shown with loading (L), flowthrough (FT), elution (E) fractions after treatment with P2.

The ability to enrich sialylated glycopeptides was tested by solid phase extraction of tryptic digest of bovine alpha-2-HS-glycoprotein, or fetuin bovine (Fet). This protein has been shown to display 3 *N*-glycosylation sites terminated with SA, as well as multiple *O*-glycosylation sites.^34^ Polymer P2 was incubated with the tryptic digest of Fet, with subsequent washing and elution steps. The loading and washing conditions were 80% ACN and 20% AmAc buffer (2 mM, pH = 6.0). Elution was done with 5% AA in water. The MALDI-MS spectra of the digest (L), flow through (FT) and elution (E) fractions for sialylated and desialylated fetuin are shown in Fig. 6. Peptide signals in the higher mass range (>3000 Da, mass difference *Δ* 291 Da) are known to belong to SA-containing glycopeptides (Fig. 6A).^35^ They can be observed in the loading and elution fractions, but not seen in the flowthrough. However, only part of the glycopeptides seemed to be eluted using 5% AA, with peptides having *m*/*z* > 5000 still being strongly retained on the polymer bed. Thus, simple acidic elution does not yield complete glycopeptide elution. Similar trend was observed in case of ZIC-HILIC enrichment of sialylated peptides, where additional elution steps with buffer (100 mM AmBic) or DHB are needed to completely elute high molecular weight sialylated peptides.^36^ Sialidase treatment resulted in different peptide profile with peptides < 4000 *m*/*z*, which possibly belong to desialylated or partially desialylated glycopeptides (based on the *Δ* 291 Da). Those peptides were found both in the flow through and elution fractions, reflecting a weaker interaction with the MIP (Fig. 6B). This demonstrates the importance of the sialic acid moiety for the glycopeptide retention. Other hydrophilic peptides are also found in the elution in the lower *m*/*z* range, possibly bearing *O*-glycans, primary amine and/or phosphate groups (Fig. S20†). Dephosphorylation has been proven to be a crucial step prior to SA enrichments using TiO_2_ and HILIC and might also be implemented in MIP protocols.^35^

## Conclusions

In conclusion, we have developed a high-capacity dual anion–cation imprinted receptor for selective capture of sialic acid. The polymer features an entirely neutral backbone composed of urea and crown ether monomers for simultaneous binding of anion and cation pairs in highly organic media. Comparing urea and thiourea monomers, the latter showed higher affinity towards SA, explained by its higher acidity in comparison to the urea functionality. However, imprinting was most successful with the urea monomer, possibly due to deprotonation effects of the more acidic thiourea-based monomer. In line with the combinatorial design, the polymers displayed preferential binding of SA carrying Na^+^, K^+^ and NH_4_^+^ counterions. This led to receptors showing to be compatible with glycomics applications. Preliminary solid phase extraction studies on fetuin digests suggested an exceptionally strong affinity of the polymers for sialylated peptides. Collectively, these dual ion imprinted receptors could open up some new possibilities in glycomics. (1) The neutral nature of the MIPs addresses the charge biased non-specific retention effects commonly seen with ion-exchanging stationary phases. (2) The strong glycan affinity should translate into a faster, more effective clean-up and enhanced sensitivity for low-abundant peptides. (3) Previously proven urea-MIPs targeting phosphopeptides may be combined with these SA-MIPs for simultaneous enrichment of two PTMs. Our current efforts aim at integrating these MIPs in existing glycomics workflows for rapid analysis of intact sialylated glycopeptides.

## Experimental part

### Materials

*N*-Acetylneuraminic acid (SA or Neu5Ac), 2,6′-sialyllactose (6SL) and 2,3′-sialyllactose (3SL) sodium salts were purchased from Carbosynth Limited. d-Glucuronic acid (GA) was received from Fluka. Ethylene glycol dimethacrylate (EGDMA) and 18-crown-6 (18C6) was from Acros Organics. *N*,*N*′-Azo-bis(2,4-dimethyl)valeronitrile (ABDV) was purchased from Wako Chemicals GmbH (Neuss, Germany). Acetic acid, phenol, sulfuric acid, ammonium acetate, ammonium bicarbonate, formic acid, trifluoroacetic acid (TFA), anhydrous methanol (MeOH), DMSO-d_6_ and methanol-d_4_ (CD_3_OD) were from VWR chemicals. 4-Vinylbenzo-18-crown-6 (VB18C6) was purchased from Ambinter (Orléans, France). 4-Vinylaniline, 3,5-bis(trifluoromethyl)phenyl isothiocyanate and 3,5-bis(trifluoromethyl)phenyl isocyanate were obtained from Sigma-Aldrich. Neuraminidase type VIII from *Clostridium perfringens* (5 units) was from Sigma. 2-AB labelled SL was synthesized according to a procedure reported before,^37^ with purification by column chromatography. EGDMA was passed through a column of activated basic alumina to remove inhibitor and stored at −20 °C before polymerization. All solvents for HPLC analysis were HPLC grade and were purchased from VWR. The (thio)urea functional monomers were synthesized as reported before.^38^

### Instruments and methods

^1^H NMR spectra were recorded on an Agilent Mercury 400 MHz instrument. High performance liquid chromatography (HPLC) measurements were carried out on an Alliance 2795 instrument equipped with 2996 PDA detector (Waters, Milford, MA, USA). Mass spectra were recorded on a Waters QUATTRO-ZQ Spectrometer with 2696/2795 HPLC Separations Module. Infrared spectra were recorded using a Thermo Nicolet Nexus 6700 instrument (Thermo Scientific, Waltham, MA, USA). UV absorbance measurements were performed on a Safire plate reader (Tecan Group Ltd., Männedorf, Switzerland) using a polystyrene 96-well microplate. Microwave assisted extraction was achieved using a Biotage Initiator 2.5+ Microwave system with Robot Sixty from Biotage AB (Uppsala, Sweden).

### ^1^H NMR spectroscopic titrations

All ^1^H NMR spectroscopic titrations were performed in dry DMSO-d_6_ and CD_3_OD. An increasing amount of guest (SA·Na or SA·Na18C6) was titrated into a fixed amount of functional monomers FM1 and FM2. The concentration of the monomer was 2 mM and the amount of added guest was 0, 0.25, 0.5, 0.75, 1.0, 1.5, 2.0, 4.0, 6.0, and 10.0 equivalents. The complexation induced shifts (CISs) of relevant protons were followed and titration curves were constructed of CIS *versus* guest concentration (*c*). The raw titration data were fitted to a 1 : 1 binding site model (eqn (1)):1
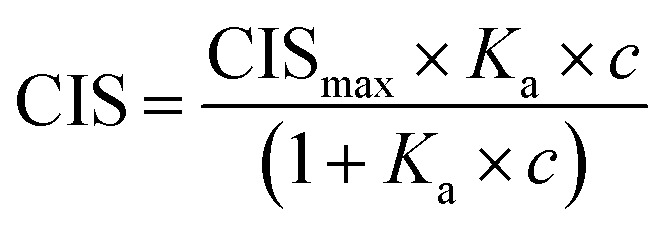
where CIS_max_ is the maximum CIS at saturation, *K*_a_ is the association constant. The fitting was performed by nonlinear regression using GraphPad Prism v.9.0 (GraphPad Software, USA).

### Polymer synthesis

The following general procedure was used for preparing imprinted polymers P1–P6. Templates SA·Na or GA·Na (0.05 mmol) were incubated with the FM1 or FM2 (0.05 mmol) with/without VB18C6 (0.05 mmol) in 0.6 mL dry methanol for 30 min. Then EGDMA (1 mmol) was added to the template/monomer mixture. The initiator ABDV (1 mol% from polymerizable double bonds) was added to the solution. Mixture was cooled to 0 °C on ice and purged by a flow of dry nitrogen for 5 min. Polymerization was initiated by placing the sealed vials into a water bath heated to 50 °C for 24 h. Afterwards, the polymers were crushed, sieved with 25–50 mesh sieves and subjected to template removal. Non-imprinted polymers (NIP) P_N_1–P_N_5 were prepared in the same manner described above, but with the omission of the template from the pre-polymerization solution.

### Template removal

Polymers were first washed with MeOH (1 × 10 mL), with MeOH/0.1 M HCl 1 : 1 v/v (3 × 10 mL) with 1 h incubation on a shaker. Microwave assisted template extraction was performed with FA–MeOH 1 : 1 v/v (7 × 5 mL) at 100 °C for 1 h. Afterwards, polymers were washed with 3 × 10 mL MeOH/H_2_O 1 : 1 v/v, and, finally, 2 × 10 mL methanol and dried *in vacuo*. All wash fractions were analyzed by HPLC-UV for template removal.

### Template binding tests

Polymers (5 mg each) were suspended in 0.5 mL of a mixture of SA, SA·K, SA·Na, SA·TBA, SA·PMP or SA·NH_4_ (each 0.5 mM) in 100% MeOH and shaken for 24 h at RT. Salts were prepared by reaction of SA with the corresponding bases and subsequent solvent evaporation. Effect of water content on binding was tested with 0.5 mL of 0.5 mM SA·Na in 10–100% MeOH–H_2_O v/v. For binding isotherms, polymers (5 mg) were incubated with 0.5 mL of SA·Na in 95% MeOH (0.125, 0.250, 0.5, 0.75, 1.0, 1.25, 1.5, 1.75 and 2.0 mM) for 24 h at RT. Afterwards, the samples were centrifuged and the supernatant (0.2 mL) was dried (Genevac EZ-2 evaporator), redissolved in 0.2 mL of 75% ACN 25% ammonium acetate buffer (10 mM pH = 6.0) and analyzed by HILIC HPLC using PolyHYDROXYETHYL A (PolyLC Inc, 3 μm, 100 Å, 100 × 3.2 mm). Mobile phases were (A) ACN and (B) ammonium acetate buffer (10 mM, pH = 6.0). An isocratic method of 75% B and 25% B at a flow rate of 0.5 mL min^−1^ was used. The injection volume was 10 μL and the detection was performed by UV absorbance measurement at 205 nm (345 nm for SL-AB). Each experiment was performed in triplicate.

The resulting peak areas were used to calculate the bound percent of analyte (Bound%) on the polymer according to eqn (2):2
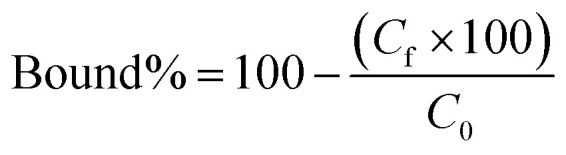
where *C*_0_ is the initial solute concentration, *C*_f_ is the final free solute concentration in the supernatant.

Amount of bound analyte per mass of polymer (*B*, μmol g^−1^) was calculated according to eqn (3) and plotted against *C*_f_.3
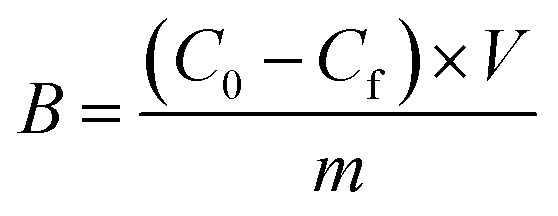
where *V* is total volume of the analyte solution and *m* is polymer mass.

The resulting binding isotherm was fitted to one site specific binding site model (eqn (4)):4
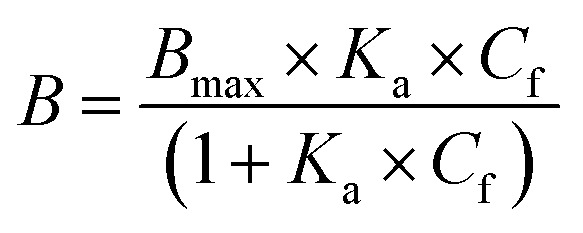
where *B*_max_ is the maximum amount of solute bound by polymer at saturation, *K*_a_ is the association constant. The fitting was performed by nonlinear regression using GraphPad Prism v.9.0 (GraphPad Software, USA).

### Glucuronate binding test

Polymers (5 mg) were incubated with 0.5 mL of GA·Na solutions (0.125, 0.250, 0.5, 0.75, 1.0, 1.25, 1.5, 1.75 and 2.0 mM) in 95% MeOH for 24 h at RT. The supernatants were dried and redissolved in water. The phenol–sulfuric colorimetric assay was then used to measure carbohydrate concentrations in the supernatant.^39^ First, 25 μL of 5 wt% phenol was added to 25 μL of aqueous carbohydrate analyte solution previously aliquoted into the microplate, followed by mixing with a pipettor. Next, 150 μL of H_2_SO_4_ was added to each well and mixed with a pipettor. Solutions were incubated for 15 min at 80 °C. After cooling to room temperature, the absorbance was measured at 490 nm using a microplate reader.

### Labeled sialyllactose binding

Binding of 2AB labeled sodium salts of sialyllactoses (3SL-AB and 6SL-AB) was tested in 80% MeOH or ACN with 20% of different modifiers: 0.5 vol% FA, ammonium formate (AmFor, 10 mM pH 4), ammonium acetate (AmAc, 10 mM pH 6), ammonium bicarbonate (AmBic, 10 mM, pH 8). Polymers (5 mg each) were suspended in 0.5 mL of an equimolar mixture of 3SL-AB and 6SL-AB (each 50 μM) and shaken for 24 h at RT. Afterwards, the samples were centrifuged and the supernatant (0.2 mL) was dried (Genevac EZ-2 evaporator), redissolved in 0.2 mL of 80–20 vol% ACN–ammonium acetate (10 mM pH = 6.0) and analyzed by HILIC HPLC using PolyHYDROXYETHYL A (PolyLC Inc, 3 μm, 100 Å, 100 × 3.2 mm). Mobile phases were (A) ACN and (B) ammonium acetate buffer (10 mM, pH = 6.0). An isocratic method of 80% B and 20% B at a flow rate of 0.5 mL min^−1^ was used. The injection volume was 10 μL and the detection was performed by UV absorbance measurement at 255 nm.

### Glycopeptide enrichment tests

A tryptic digest of bovine fetuin was prepared according to a reported procedure.^35^ Shortly, 1 mg of fetuin bovine was dissolved in 200 μL of AmBic (50 mM, pH 7.8) containing 10 mM DTT. Solution was incubated 30 min at 60°. Afterwards, protein was alkylated with 40 mM iodoacetamide for 1 h at room temperature in dark. After quenching the reaction with addition of 10 mM DTT, solution was digested using trypsin (20 μg) at 37 °C for 12 h. A negative control of sialidase treated protein has been also prepared by treating digest with 0.005 units of neuraminidase overnight at 37 °C in AmAc (50 mM, pH 6).

Aliquots of the digest (2 nmol) were dried (Genevac EZ-2 evaporator) and redispersed in 1 mL of the loading buffer prior enrichment. Polymers (2 mg) were first equilibrated with loading buffer in an Eppendorf tube. After removing the supernatant, 100 μL of digest (200 pmol) was loaded onto the polymer bed and incubated on a shaker for 1 h. Supernatant was collected and polymers were rinsed with 100 μL of loading buffer for 5 min. Loading and washing fractions were pooled together. Elution was done with 100 μL of 5 vol% acetic acid. Collected fractions were dried and redispersed in 20 μL of 0.1% TFA. Afterwards, 1.5 μL of each fraction was spotted on MALDI plate, followed by 1.5 μL of matrix solution (DHB in 50/50 ACN/H_2_O 0.1% TFA), dried and analyzed in linear positive mode. Average of 2000 spectra were collected in 1000–8000 *m*/*z* range.

## Author contributions

The first draft of the manuscript was written by LM and subsequent versions by LM and BS jointly. LM and EV performed the experimental work reported. All authors have given approval to the final version of the manuscript.

## Conflicts of interest

There are no conflicts to declare.

## Supplementary Material

RA-011-D1RA06962D-s001
