# Supplementary material for: Combinatorial design of a sialic acid imprinted binding site exploring a dual ion receptor approach
Source: RSC Adv. 2021 Oct 22;11(54):34329–37. doi: 10.1039/d1ra06962d (PMC9042400; doi:10.1039/d1ra06962d)
Supplement: RA-011-D1RA06962D-s001 [file RA-011-D1RA06962D-s001.pdf]

Electronic Supplementary Material (ESI) for RSC Advances.
This journal is © The Royal Society of Chemistry 2021

Supporting Information

Combinatorial design of a sialic acid imprinted binding site exploring a dual ion receptor approach

Mavliutova L.,[a] Verduci E.,[a] Sellergren B.[a,*]

[a] Department of Biomedical Sciences, Faculty of Health and Society, Malmö University, SE-20506 Malmö, Sweden. E-mail: borje.sellergren@mau.se

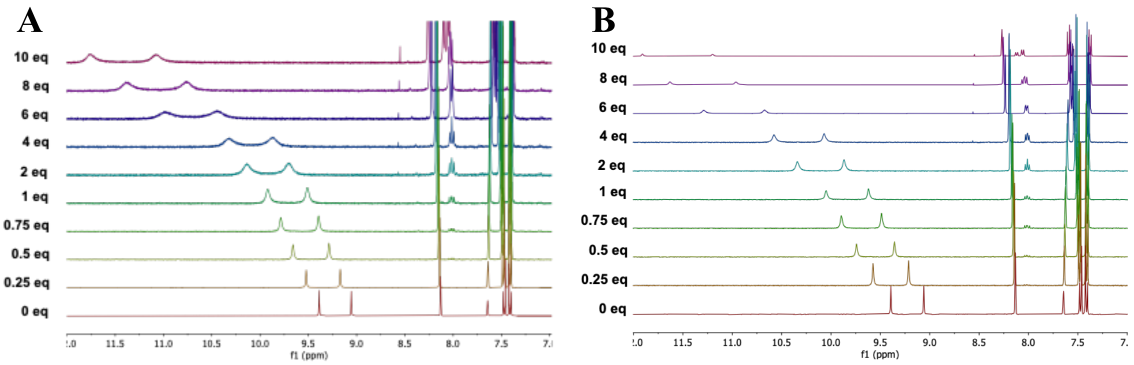

Figure S1. ^{1}H NMR spectra of FM1 with increasing amount of SA·Na (A) and SA·Na18C6 (B) in DMSO-d_6.

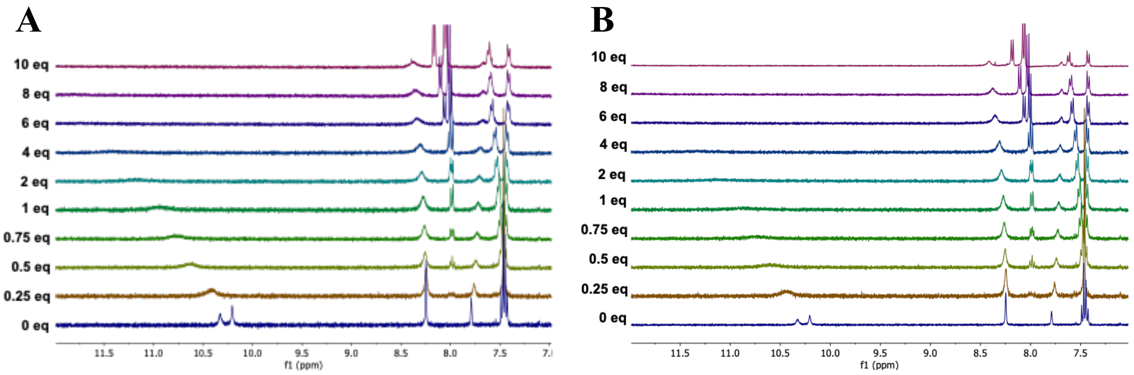

Figure S2. ^{1}H NMR spectra of FM2 with increasing amount of SA·Na (A) and SA·Na18C6 (B) in DMSO-d_6.

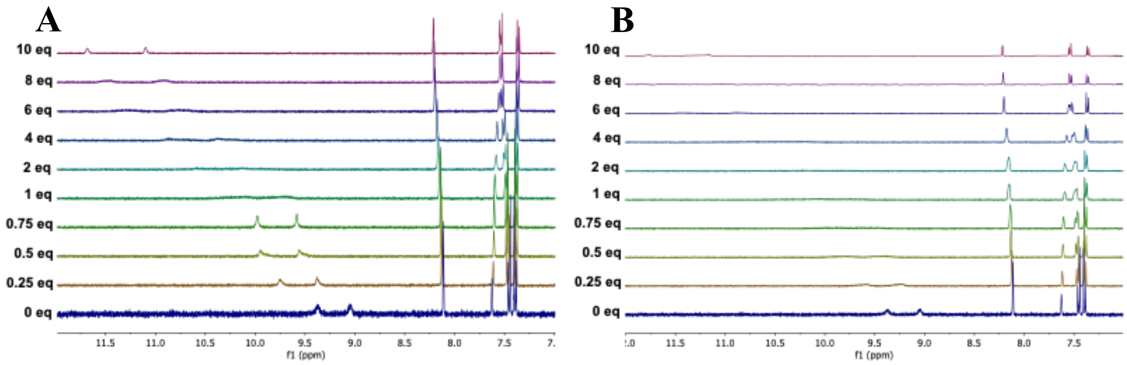

Figure S3. ^{1}H NMR spectra of FM1 with increasing amount of GA·Na (A) and GA·Na18C6 (B) in DMSO-d_6.

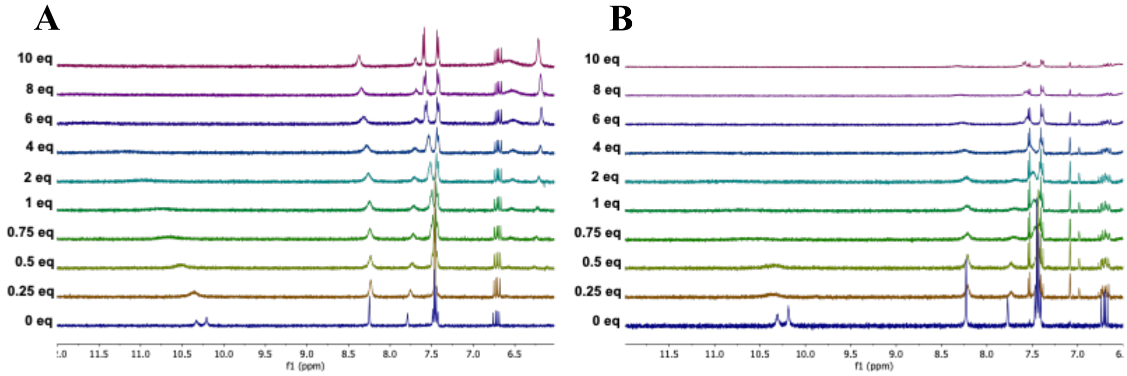

Figure S4. ^{1}H NMR spectra of FM2 with increasing amount of GA·Na (A) and GA·Na18C6 (B) in DMSO-d_6.

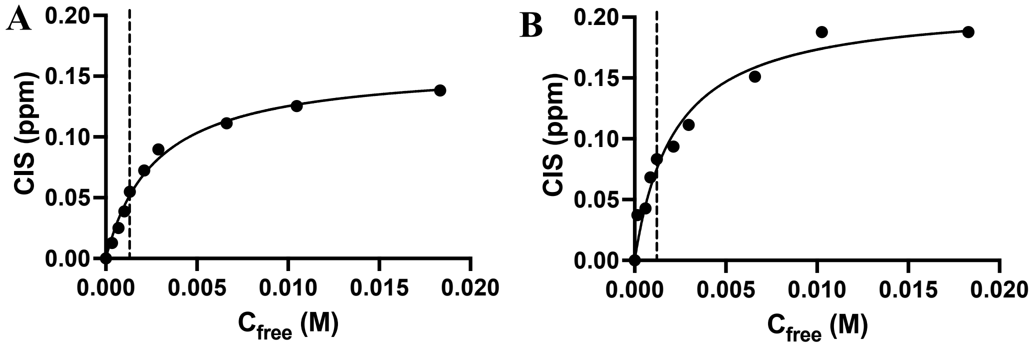

Figure S5. Complexation induced shifts of H_a protons of FM2 upon addition of GA·Na (A) and GA·Na18C6 (B) in DMSO-d_6, fitted to one site specific binding isotherm.

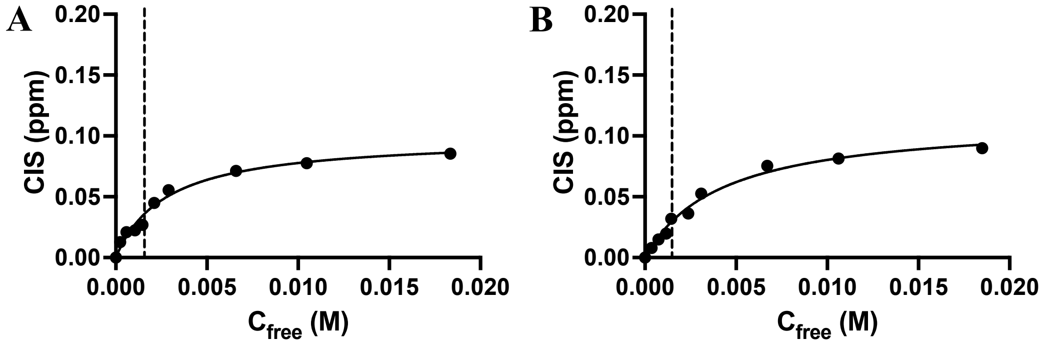

Figure S6. Complexation induced shifts of H^a protons of FM1 upon addition of GA·Na (A) and GA·Na18C6 (B) in DMSO-d_6, fitted to one site specific binding isotherm.

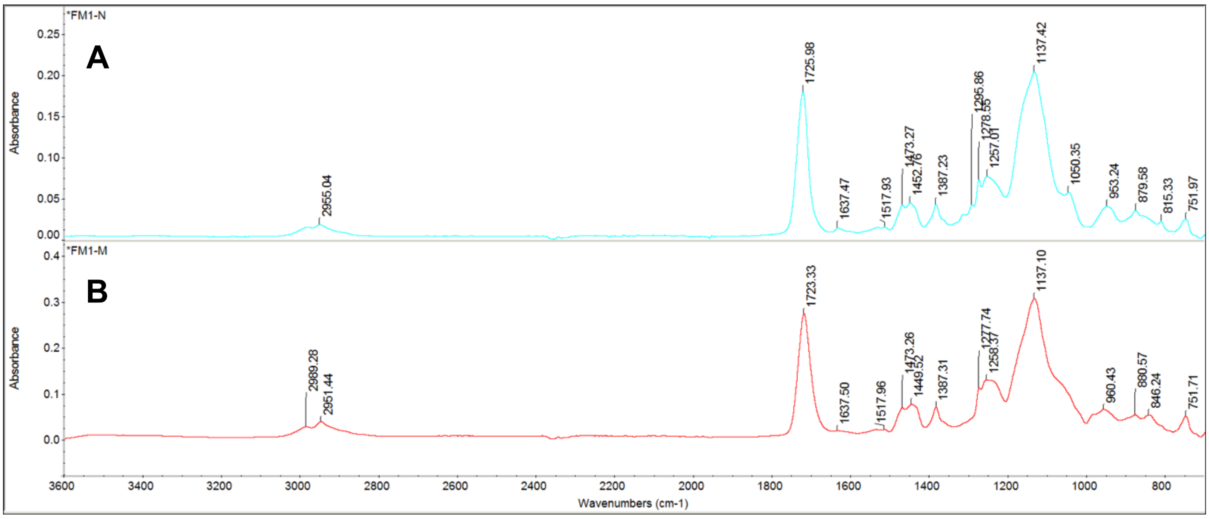

Figure S7. FTIR spectra of **P$_N$1** (A) and **P1** (B) polymers.

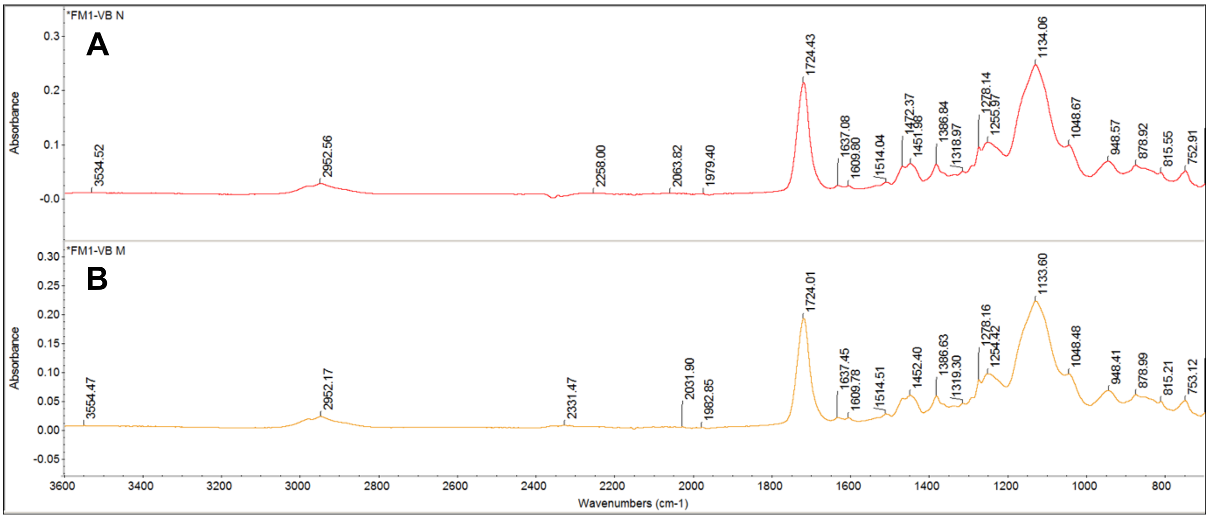

Figure S8. FTIR spectra of **P$_N$2** (A) and **P2** (B) polymers.

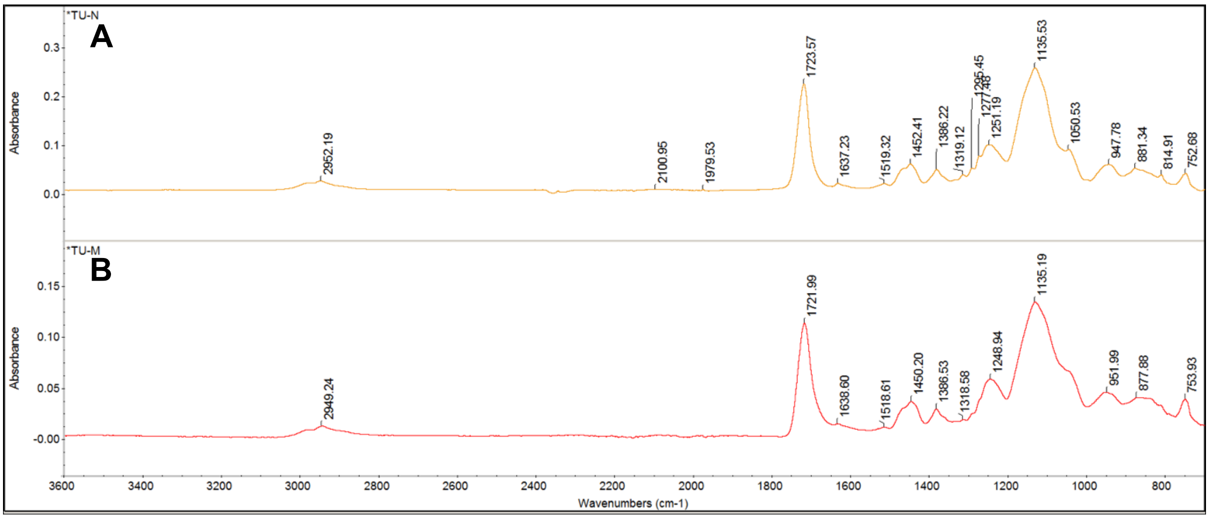

Figure S9. FTIR spectra of $\mathbf{P_N3}$ (A) and **P3** (B) polymers.

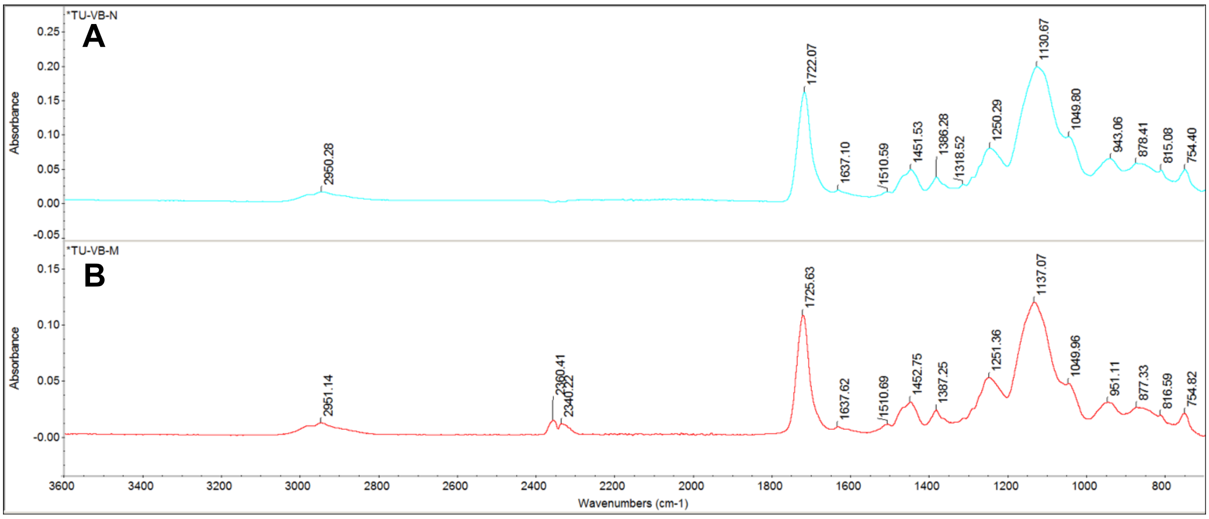

Figure S10. FTIR spectra of $\mathbf{P_N4}$ (A) and **P4** (B) polymers.

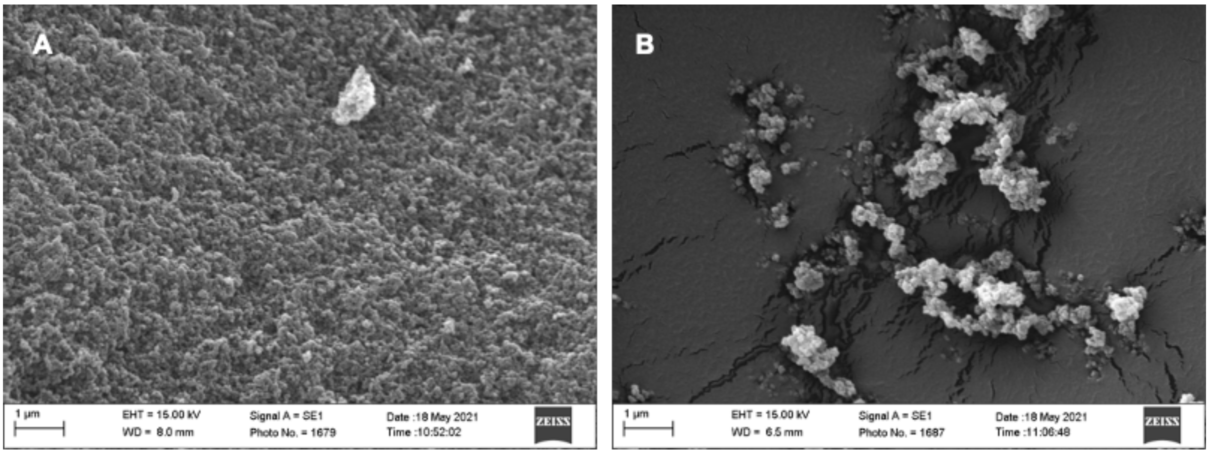

Figure S11. SEM images of **P1** (A) and **P_N1** (B) at x25000 magnification.

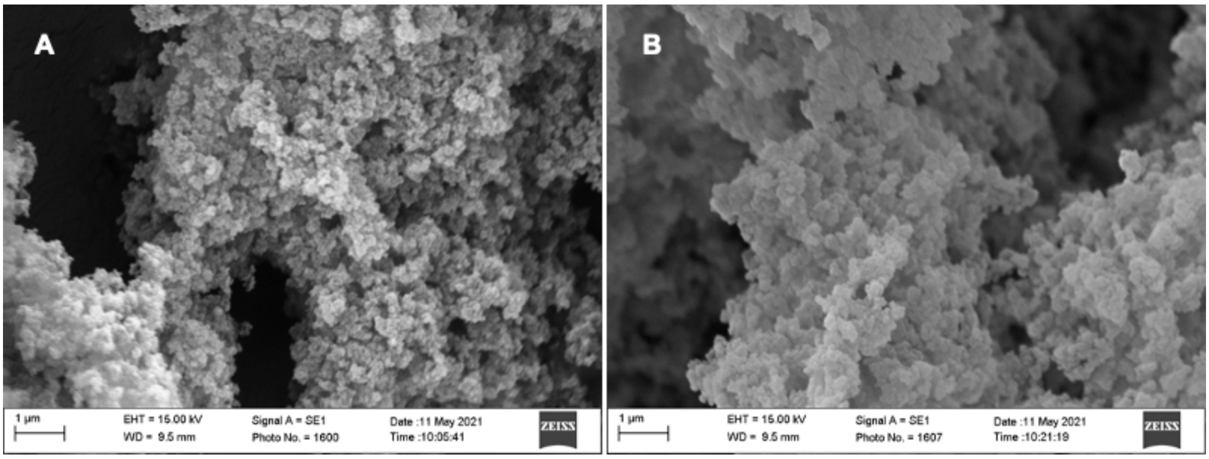

Figure S12. SEM images of **P2** (A) and **P_N2** (B) at x25000 magnification.

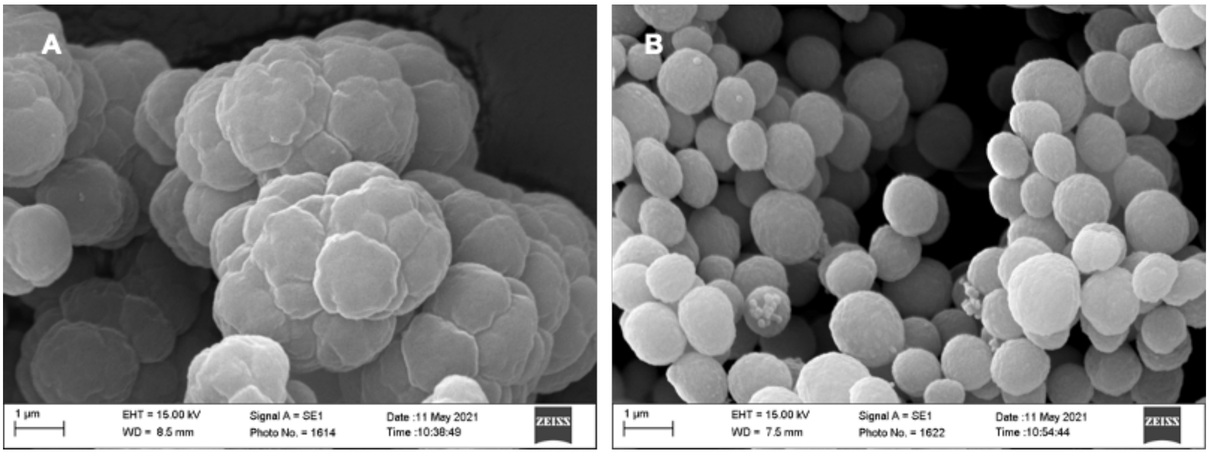

Figure S13. SEM images of **P3** (A) and **P$_N$3** (B) at x25000 magnification.

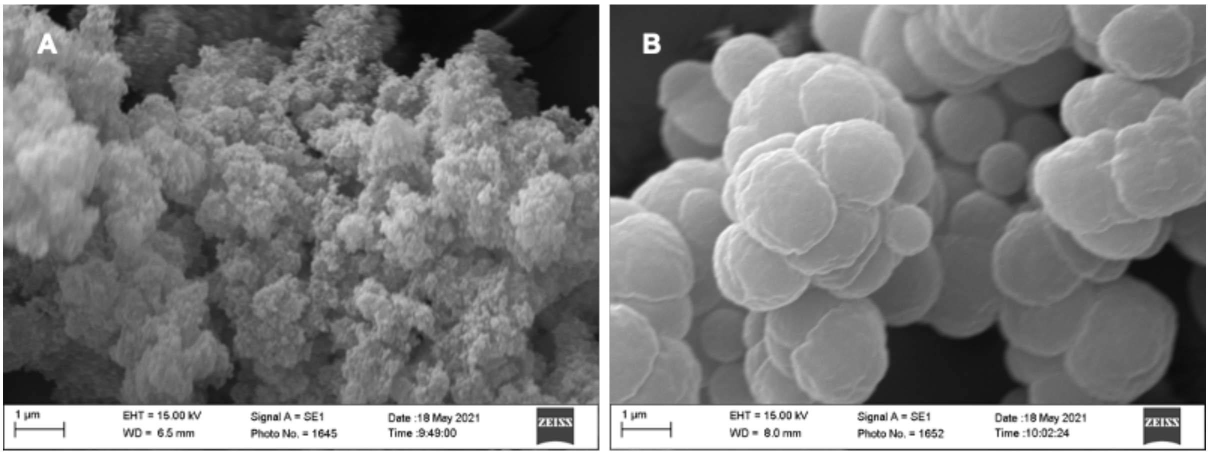

Figure S14. SEM images of **P4** (A) and **P$_N$4** (B) at x25000 magnification.

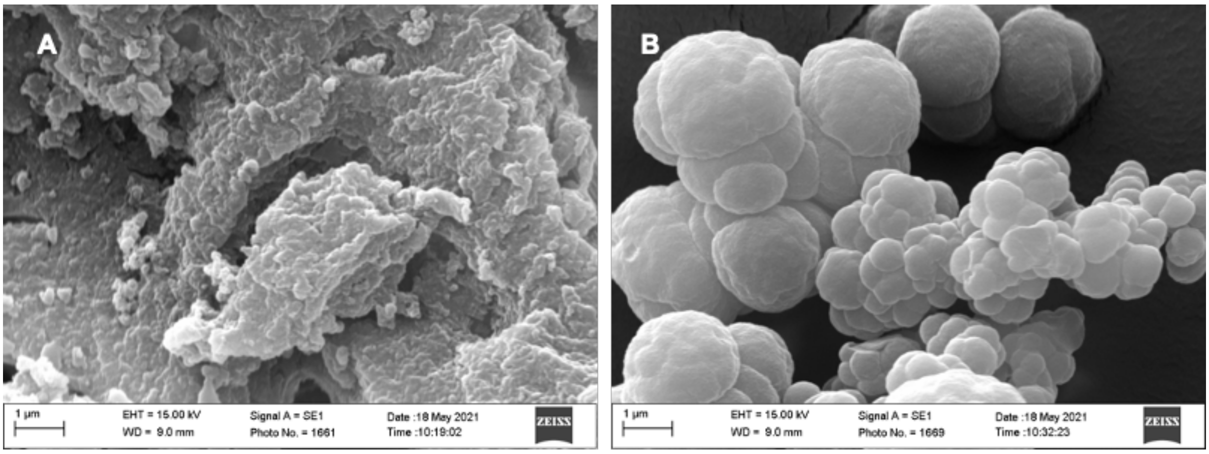

Figure S15. SEM images of **P5** (A) and **P$_N$5** (B) at x25000 magnification.

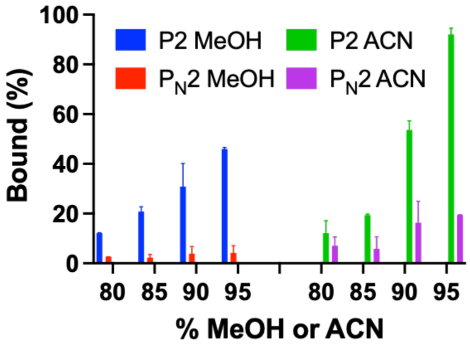

Figure S16. Batch binding of control polymers **P2**/**P$_N$2** with 0.5 mM SA·Na in 80-95% MeOH and 80-95% ACN.

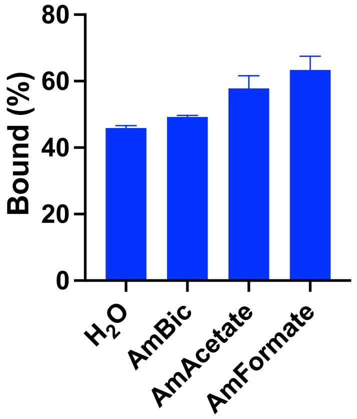

Figure S17. SA·Na-MIP **P2** batch binding with 0.5 mM SA·Na in 95% MeOH with 50 mM ammonium buffers – bicarbonate, acetate and formate.

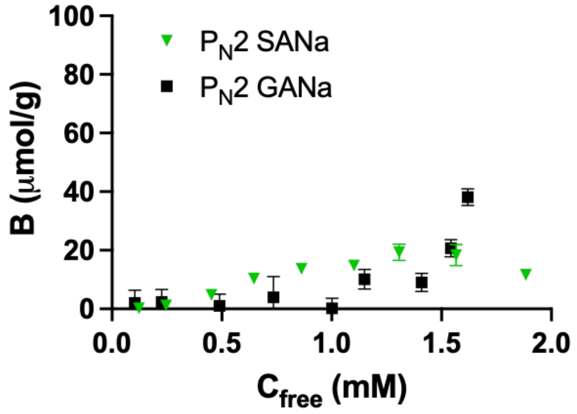

Figure S18. Binding isotherms of non-imprinted polymer **P$_N$2** with GA·Na in 95% MeOH.

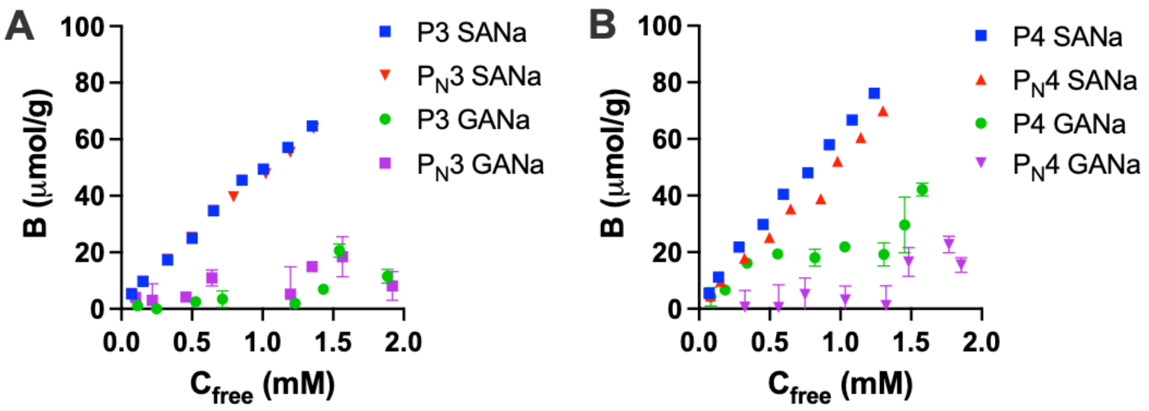

Figure S19. Binding isotherms in 95% MeOH with SA·Na and GA·Na for thiourea-based polymers **P3/P$_N$3** (A) and **P4/P$_N$4** (B).

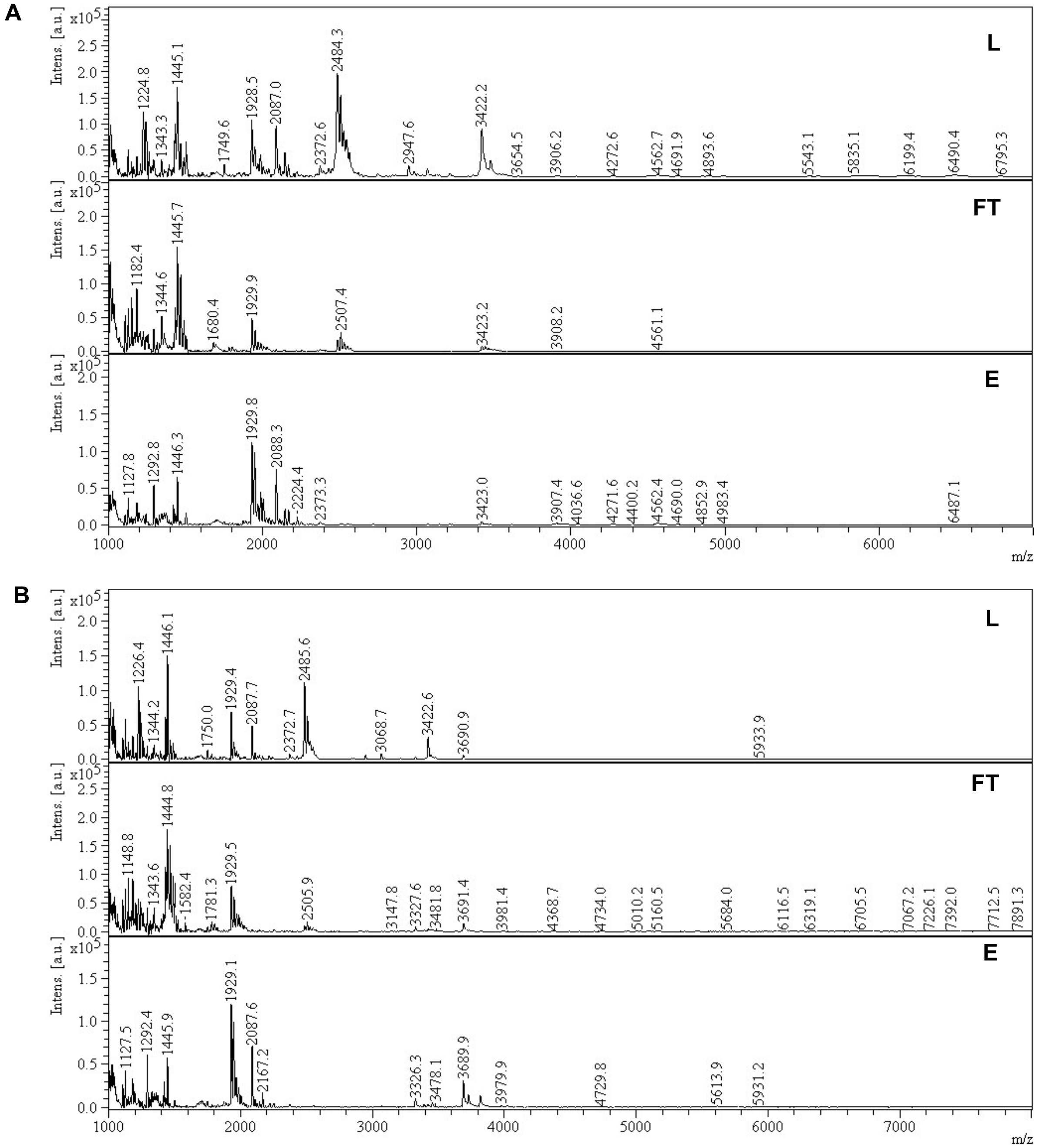

Figure S20. MALDI-TOF-MS of tryptic peptide mixture of fetuin bovine (A) and sialidase treated fetuin bovine (B). General peptide spectrum with loading (L), flowthrough (FT), elution (E) fractions after incubation with **P2**.

Table S1. Values obtained from fitting binding isotherm of **P2** with SA-Na and GA-Na in 95% methanol to one site specific binding isotherm.

	SA•Na	GA•Na
One site -- Specific binding [2]		Interrupted
Best-fit values		
Bmax	76.44	n/a
Ka	2.650	n/a
Std. Error		
Bmax	3.659	
Ka	0.3673	
95% CI (asymptotic)		
Bmax	68.59 to 84.29	
Ka	1.862 to 3.438	
Goodness of Fit		
Degrees of Freedom	14	
R squared	0.9674	
Sum of Squares	133.0	
Sy.x	3.082	

Table S2. Values obtained from fitting binding isotherm of **P6** with SA-Na and GA-Na in 95% methanol to one site specific binding isotherm.

	SA•Na	GA•Na
One site -- Specific binding [2]	Interrupted	
Best-fit values		
Bmax	n/a	77.36
Ka	n/a	1.381
Std. Error		
Bmax		15.26
Ka		0.5994
95% CI (asymptotic)		
Bmax		41.28 to 113.4
Ka		-0.03672 to 2.798
Goodness of Fit		
Degrees of Freedom		7
R squared		0.9138
Sum of Squares		203.9
Sy.x		5.397
